# Impact of heart motion on radiation dose in the heart and left ventricular myocardium during breast cancer treatment

**DOI:** 10.3389/fonc.2025.1503131

**Published:** 2025-06-25

**Authors:** Zhiqing Xiao, Yanqiang Wang, Miao Wang, Han Guo, Xiaotong Lin, Lei Tian, Junling Liu, Xiuwu Li, Xiaoying Xue

**Affiliations:** ^1^ Department of Radiation Oncology, the Second Hospital of Hebei Medical University, Shijiazhuang, Hebei, China; ^2^ Hebei Key Laboratory of Etiology Tracing and Individualized Diagnosis and Treatment for Digestive System Carcinoma, Second Hospital of Hebei Medical University, Shijiazhuang, Hebei, China

**Keywords:** breast cancer, radiotherapy, deep inspiration breath-hold technique, descending coronary artery, cardiac dose

## Abstract

**Objective:**

This study aimed to investigate changes in the geometric position and dosimetry differences of the heart and the left anterior descending coronary artery (LAD) during radiotherapy with deep inspiration breath hold (DIBH) in patients with left-sided breast cancer after radical mastectomy.

**Methods:**

A retrospective analysis was undertaken on 10 patients with left-sided breast cancer who received DIBH radiotherapy. Changes in the motion position of the heart and the LAD and dosimetric differences were compared by analyzing the planning CT and cone beam CT (CBCT) images.

**Results:**

Heart volume was negatively correlated with the heart *V*_5_ and *D*_mean_ and positively correlated with *V*_30_ and *D*_max_. Changes in the heart volume were positively correlated with the dose changes in both the heart and the LAD. The lateral (*X*-axis) motion of the heart was positively correlated with the heart *V*_15_ and *V*_20_ and the LAD *D*_max_, but negatively correlated with the heart *D*_max_. Superior–inferior (*Y*-axis) motion was negatively correlated with the heart *V*_15_, *V*_20_, *V*_30_, and *D*_mean_ and changes in the LAD dose. Anterior–posterior (*Z*-axis) motion was positively correlated with changes in both the heart dose and the LAD *V*_5_, *V*_15_, and *V*_20_ doses. Owing to alterations in the centroids, the heart requires expansions of the planning margins of 1.33, 4.10, and 2.42 mm in the *X*, *Y*, and *Z* directions, respectively, while the LAD requires expansions of 3.13, 1.79, and 5.43 mm in the corresponding directions. The distances of the cardiac boundary boxes during the different sessions showed a positive correlation with the heart *V*_5_, *V*_15,_ *V*_20_, *V*_30_, and *D*_mean_ and a negative correlation with the LAD *V*_5_ and *D*_max_.

**Conclusions:**

During the implementation of DIBH radiotherapy for the treatment of left-sided breast cancer, dose assessment for the heart and the LAD provided by static CT planning may contain some inaccuracies. Accordingly, it is recommended to reasonably adjust the organ-at-risk external boundaries in the treatment plan to effectively control the doses received by the heart and the LAD, thereby ensuring patient safety.

## 1 Introduction

The incidence of and the mortality from female breast cancer rank first and fifth, respectively, among malignant tumors in women in China, posing a serious threat to their physical and mental health (1). Radiotherapy plays an important role in the comprehensive treatment of breast cancer through its ability to effectively reduce the local tumor recurrence rate and improve patient survival (2). Precision radiotherapy technologies such as intensity-modulated radiation therapy (IMRT), volumetric modulated arc therapy (VMAT), and image-guided radiation therapy (IGRT) are now widely used. It is increasingly clear that the toxic side effects and long-term risks associated with radiation therapy impact the overall survival rate of patients with breast cancer. Notably, epidemiological studies have demonstrated that the incidence of major cardiovascular events is significantly elevated among patients with left-sided breast cancer receiving radiation therapy compared with patients with right-sided breast cancer receiving comparable treatment. This is attributable to the heart’s anatomical proximity to the left breast cavity, which results in it receiving greater radiation exposure. These observations suggest that radiotherapy can cause long-term heart disease. The heightened risk of radiation-induced cardiotoxicity (3) stems from the spatial relationship between the left breast and cardiac structures. During expiration, the posteromedial aspect of the left chest wall directly abuts the pericardium overlying the left anterior descending coronary artery (LAD), creating a zone of critical dose deposition where cardiac substructures receive substantial radiation exposure. Radiation oncologists closely monitor the potential exposure risk due to this anatomical location. The deep inspiration breath hold (DIBH) technique moves the heart away from the chest wall target area and may reduce the doses received by the heart and the LAD by 25%–67% and 20%–73%, respectively (4). The use of this technique helps limit the maximum and average cardiovascular radiation exposure, thereby effectively protecting the heart (5).

However, even with the application of the DIBH technique, changes in the movement of the heart itself cannot be ignored. Expansion of the protective margins in radiotherapy planning serves two primary purposes: to compensate for the cardiac displacement caused by respiratory motion (e.g., inferior and posterior shifts during inspiration) and to ensure dosimetric safety by addressing the limitations of static plans, where cardiac motion may lead to target underdosing or cardiac overdosing. Margin optimization thus balances target coverage with cardiac protection. Clinically, this strategy not only reduces cardiotoxicity risks—mitigating radiation-induced myocardial injury and coronary artery disease and thus improving long-term survival outcomes—but also enhances the tumor control rates by enabling safe dose escalation to the target volume. Furthermore, expanded margins allow for personalized adaptation strategies, tailoring the protective scope to individual respiratory patterns and tumor locations (e.g., left-sided breast cancer), thereby enhancing therapeutic delivery precision while prioritizing organ-at-risk (OAR) preservation. Therefore, in this study, we incorporated the concepts of bounding box and centroid. Although inter- and intra-fraction differences in the chest wall during DIBH are small when skeletal structures are aligned, the cardiac and LAD displacement may be greater during DIBH compared with that during free breathing. Cardiac motion-induced displacement introduces clinically significant discrepancies between planned and delivered radiation doses. In this investigation, we enrolled patients with left-sided breast cancer undergoing post-mastectomy radiotherapy with DIBH, establishing safety margins based on the cardiac motion parameters derived from centroid trajectory analysis. Through time-resolved dosimetric evaluation, we quantified residual dose uncertainties in cardiac substructures—particularly the LAD coronary artery—caused by intrinsic myocardial periodicity despite motion management, providing innovative strategies for precision radiotherapy through margin optimization.

## 2 Materials and methods

### 2.1 Patient selection

Data were retrospectively collected from10 female patients who underwent modified radical mastectomy for left-sided breast cancer, with each patient undergoing 25 CBCT scans, totaling 250 CBCT datasets. The patients’ ages ranged from 28 to 66 years, with a mean of 45.9 years (**Table 1**). The inclusion criteria were as follows: 1) pathologically confirmed left-sided breast cancer and indications for adjuvant radiotherapy; 2) clear cognition and awareness, good cardiopulmonary function, and stable breathing amplitude; 3) normal hearing, ability to cooperate with DIBH training, breath-holding time exceeding 20 s after deep inhalation with consistent respiratory frequency and amplitude across multiple repetitions; and 4) each patient and family member could understand the treatment process and sign an informed consent form.

**Table 1:** Patient characteristics.

Patient no.	Age (years)	BMI	TNM	Surgery (mastectomy/lumpectomy)	Hypertension	Diabetes
1	50	22.9	pT_2_N_1_M_0_	Mastectomy	No	No
2	28	21.9	pT_2_N_1_M_0_	Mastectomy	Yes	No
3	66	23.2	pT_2_N_1_M_0_	Mastectomy	No	No
4	49	17.8	pT_3_N_0_M_0_	Mastectomy	Yes	Yes
5	39	28.0	pT_1_N_1_M_0_	Mastectomy	No	Yes
6	46	21.3	pT_3_N_0_M_0_	Mastectomy	No	No
7	56	23.1	pT_1_N_1_M_0_	Mastectomy	No	No
8	32	26.3	pT_2_N_3_M_0_	Mastectomy	No	No
9	53	28.3	pT_2_N_3_M_0_	Mastectomy	Yes	No
10	40	20.4	pT_2_N_1_M_0_	Mastectomy	No	No

### 2.2 Image acquisition

For immobilization and positioning, a Philips Brilliance Big Bore™ CT simulator (Philips Healthcare, Best, Netherlands) with an 85-cm bore diameter was used. Patients were placed in the supine position on a vacuum negative pressure pad with dual-arm support. For respiratory management, the Integrated Varian RGSC system was employed for pre-scan respiratory pattern analysis (10-cycle baseline acquisition) and continuous thoracoabdominal motion tracking using infrared reflective markers. The DIBH training protocol comprised 30-min coached breathing sessions using audiovisual biofeedback. The breath-hold compliance criteria were sustained hold duration ≥20 s and amplitude stability of <3 mm deviation from the target (80%–90% vital capacity). For image acquisition, a dual-phase CT acquisition protocol was used, which involved free-breathing CT (3-mm slice thickness, 120 kV, captured over three full respiratory cycles) and DIBH-CT (gated acquisition using a 3-mm amplitude window, laser alignment verification with cross-plane fiducial markers, and synchronized with RGSC tracking at 80% inspiration phase). To establish a reproducible and stable breathing amplitude during DIBH, each patient underwent 30 min of breathing training with the RGSC system before the CT simulation. While immobilized in the treatment position, using auto-visual guidance, the patients followed voice instructions to inhale deeply and hold their breath for >20 s, keeping the chest and the abdomen motionless. Patients controlled their breathing amplitude using visual feedback on the display pad. The operator established a stable breath-holding amplitude target of 80%–90% of each patient’s maximum breathing amplitude. Patients were instructed to maintain their breath hold within this range after deep inhalation. The breathing amplitude was kept as consistent as possible, with fluctuations under 2 mm. Training was repeated until the patient’s breathing amplitude range is within the range. The breathing amplitude should be kept as consistent as possible, with fluctuations < 3 mm. Free-breathing CT and DIBH-CT scans were acquired for all patients. Firstly, a free-breathing CT scan was performed, followed by deep inhalation and breath holding. A 3-mm gating window width, comparable to the minimum mean breathing amplitude observed during free-breathing scans, was selected. In the breath-holding state, a crosshair marked the center, lead dots were affixed, and lines were drawn on both sides of the body to indicate the breath-holding position. The DIBH-CT scan was subsequently performed, and both image sets were transferred to the treatment planning system (TPS).

### 2.3 Target area, risk organ delineation, and plan design

For IMRT planning, a consistent approach was used for all patients. The same oncologist delineated the clinical target volume (CTV) and the planning target volume (PTV) following the National Comprehensive Cancer Network (NCCN) breast cancer target volume delineation guidelines. A 5-mm margin was applied from the CTV to the PTV, with the PTV maintained at least 5 mm from the skin in superficial areas. The heart and the LAD were contoured without margin by a radiologic technologist using the CT-based atlas of Feng et al. (6). The LAD was delineated in the anterior interventricular groove down to the apex of the heart under supervision from a cardiologist. An oncologist verified the delineation of the heart, which extended from the layer across the midline pulmonary trunk to the apex. The outline of the LAD ranged from the anterior and inferior edge of the left coronary artery trunk along the anterior interventricular groove to the apex (6). A dose of 50 Gy in 25 fractions over 5 weeks (one fraction per day) with 6-MV photons was prescribed, and boluses were applied to account for tissue heterogeneity. All plans adhered to the ICRU 62 (1999) guidelines. The IMRT plan for each patient was created using the Varian Eclipse v.15.6 TPS to ensure that 95% of the PTV receive the prescribed dose. A pair of opposing tangential primary fields was supplemented by a pair of complementary beams angled at <15° to the tangents forming a butterfly configuration, along with matched anterior–posterior fields covering the supraclavicular fossa. Internal mammary nodal irradiation was added contingent upon target volume coverage requirements. The OAR constraints were: affected lung, *V*_20_ < 30%, *V*_30_ < 20%; spinal cord maximum dose, <45 Gy; and heart, *V*_30_ < 10%. The same physicist generated all treatment plans using six to eight beams. The Acuros XB algorithm was used for dose calculation with a 3-mm × 3-mm calculation grid. The bounding box is a 3D rectangular volume aligned with imaging axes that fully enclosed a target structure, defined by its minimum and maximum coordinates in all dimensions. The centroid, on the other hand, refers to the geometric center point of a structure, calculated as the average spatial position of all its voxels. In radiotherapy, bounding boxes quantify the extent of organ motion, while centroids track the displacement trajectories. Regarding the relationship between them, a constant bounding box volume in the presence of a significant centroid displacement indicates a motion pattern dominated by translational movement. Conversely, weak correlations between the bounding box volumetric changes and the centroid displacement suggest intrinsic tissue deformation. By computing the time-lagged cross-correlation function between the centroid trajectory coordinates and the bounding box dimensions (length/width/height), the phase-dependent characteristics of motion patterns can be quantified, enabling the identification of systole–diastole transitions or respiratory-coupled displacements.

### 2.4 Image fusion and parameter extraction

**Figure 1:**
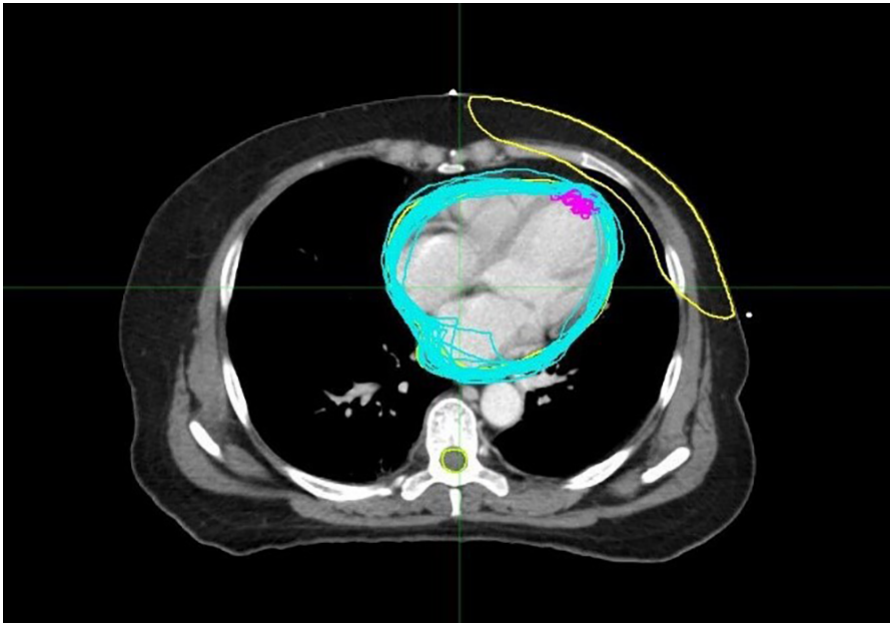
The *blue lines* represent the outlines of the heart traced in each cone beam CT (CBCT) image, while the *purple lines* represent the outlines of the left anterior descending coronary artery (LAD) traced in each CBCT.

Image-guided positioning using the DIBH technique was performed before radiotherapy delivery with the TrueBeam STx system (Varian Medical Systems, Palo Alto, CA, USA). The treating physician contoured the heart and the LAD on the deep inhalation breath-holding CBCT scan images acquired for each fraction. For this study, the MIM software facilitated rigid registrations between the planning CT images and the CBCT datasets. These registrations prioritized the accuracy of the anatomical landmarks near the PTV, such as the clavicular and costal structures, as well as the skin contours, with manual adjustments made when needed. After alignment, the heart and the LAD were recontoured on the CBCT scans and the OAR doses recalculated to account for positional variations. Quantitative analysis included translational deviations (Δ*X*, Δ*Y*, and Δ*Z*) and volumetric changes in the cardiac/LAD structures across fractions; dosimetric evaluation of the cardiac parameters (*V*_5_, *V*_15_, *V*_20_, *V*_30_, *D*_mean_, and *D*_max_) and the LAD-specific metrics (**Figure 1**); and correlation analysis between the 3D cardiac displacement vectors (derived from the center-of-mass trajectories) and the dose distribution parameters, as well as between the organ motion magnitude (quantified via the bounding box dimensions) and the radiation exposure metrics.

### 2.5 Statistical analysis

The setup errors and dosimetric parameters from 250 CBCT datasets across 10 patients were statistically processed using SPSS version 26.0 (IBM Corp., Armonk, NY, USA). Normally distributed data are presented as the mean ± standard deviation. Linear associations between the motion parameters and the dose metrics were quantified using Pearson’s correlation coefficients (two-tailed). *P*-values <0.05 were considered statistically significant.

## 3 Results

### 3.1 Correlations between heart volume and dose

**Figure 2:**
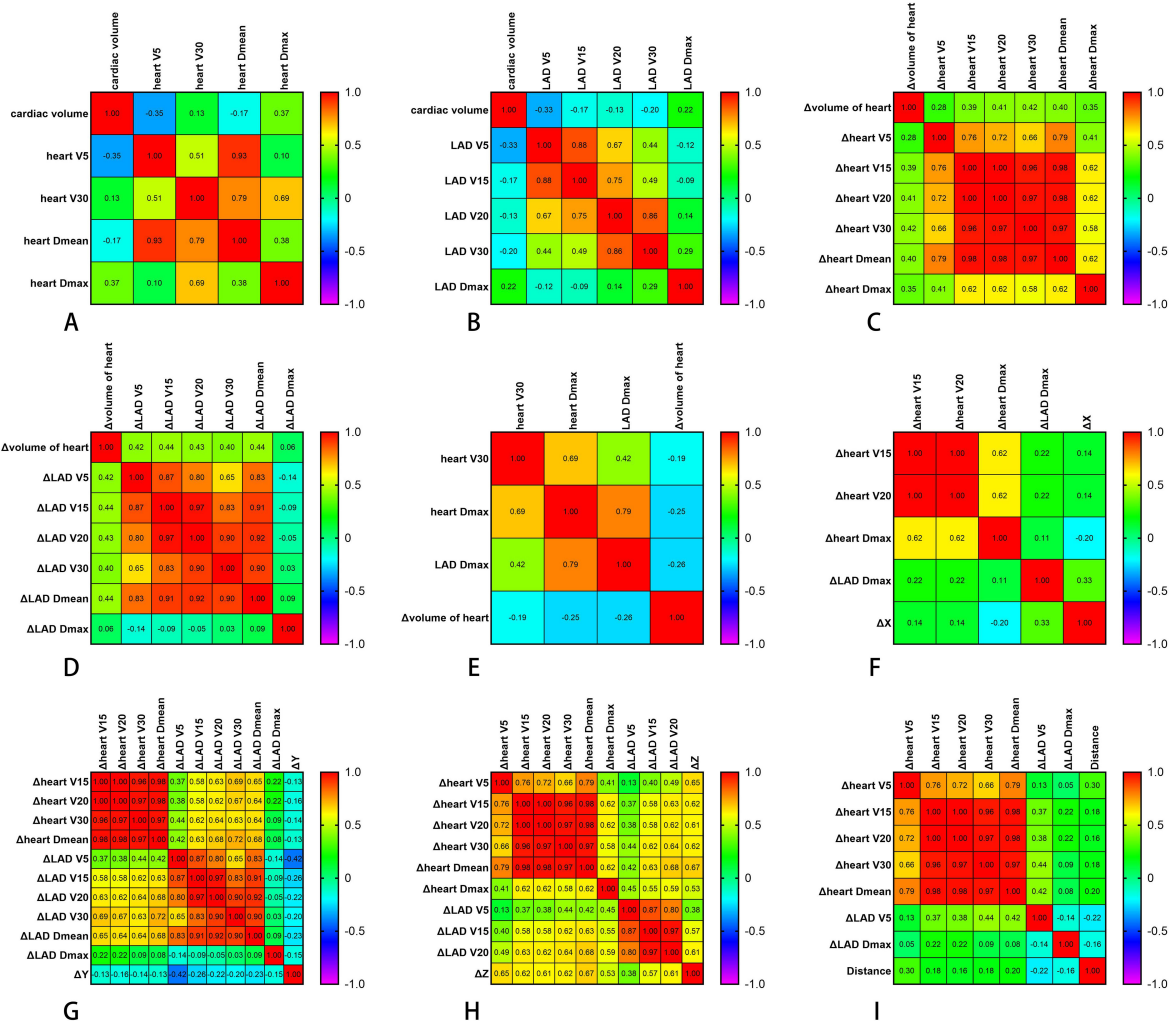
Correlations between different parameters. **(A)** Correlations between the cardiac volumes (in cubic centimeters) and the cardiac dose parameters (in centigray). **(B)** Correlations between the different cardiac volumes (in cubic centimeters) and the LAD dose parameters (in centigray). **(C)** Correlations between the heart volume changes (in cubic centimeters) and the heart dose changes (in centigray) at different times. **(D)** Correlations between the heart volume changes (in cubic centimeters) and the descending coronary artery (LAD) dose changes (in centigray) at different times. **(E)** Correlations between changes in the cardiac volume (in cubic centimeters) at different times and the cardiac and LAD dose parameters (in centigray). **(F, G)** Correlations between heart displacement in the *X* **(F)** and *Y* **(G)** directions (in centimeters) and dose change (in centigray). **(H)** Correlations between heart displacement in the *Z* direction (in centimeters) and the heart and LAD dose change (in centigray). **(I)** Correlation between the distance (in centimeters) of the different sub-boundary boxes and the dose change (in centigray). Δ denotes the difference in the dosimetric or displacement parameters between cone beam CT (CBCT) and planning CT.

Cardiac volume showed a negative correlation with the heart *V*_5_ (*r* = −0.35) and *D*_mean_ (*r* = −0.17), suggesting that larger cardiac volumes may be associated with lower *V*_5_ and mean doses to the heart, potentially favoring dose distribution optimization. Conversely, cardiac volume was positively correlated with the cardiac *V*_30_ (*r* = 0.13) and *D*_max_ (*r* = 0.37) and the LAD dose (excluding *D*_max_), indicating that an increased cardiac volume may correspond to higher values of these parameters. Notably, *V*_30_ and *D*_max_ exhibited volume-dependent variations. The presence of both inverse and positive correlations (**Figures 2A, B**) highlighted that changes in cardiac dimensions can significantly alter the dose deposition patterns during radiotherapy, potentially creating both dose-sparing and dose-escalation regions within the irradiated volume.

### 3.2 Correlations between changes in heart volume and dose

To account for variations in the cardiac volume across treatments, changes in the cardiac volume between all CBCT images and the planned CT were calculated. These changes showed moderate positive correlations with dose changes in the cardiac *V*_15_, *V*_20_, *V*_30_, *D*_mean_, and *D*_max_ (*r* = 0.39, 0.41, 0.42, 0.40, and 0.35, respectively), as illustrated in **Figure 2C**. These correlations suggest that an increase in heart volume tends to be associated with an increase in the dose received by the heart. Similarly, the change in cardiac volume was moderately correlated with the dose changes in the LAD *V*_5_, *V*_15_, *V*_20_, *V*_30_, and *D*_mean_ (*r* = 0.42, 0.44, 0.43, 0.40, and 0.44, respectively) and weakly correlated with changes in the cardiac *V*_5_ (*r* = 0.28) and the LAD *D*_max_ (*r* = 0.06), as shown in **Figure 2D**. This indicated that changes in the heart volume have a stronger positive association with lower doses to the LAD *V*_5_ and *V*_15_ than with the maximum LAD dose. The change in heart volume exhibited very weak correlations with the heart *V*_30_ (*r* = 0.186) and weak correlations with the heart *D*_max_ (*r* = 0.255) and the LAD *D*_max_ (*r* = 0.256) (**Figure 2E**). The correlation analysis revealed that the volume increases primarily affected the intermediate- to low-dose regions, with minimal impact on high-dose regions. This phenomenon may have resulted from the volumetric variations induced by cardiac motion displacing portions of the high-dose areas beyond the central beam axis.

### 3.3 Correlations of the displacement of the heart in the left and right (*X*), head and feet (*Y*), and front and back (*Z*) directions with the dose

**Figure 3:**
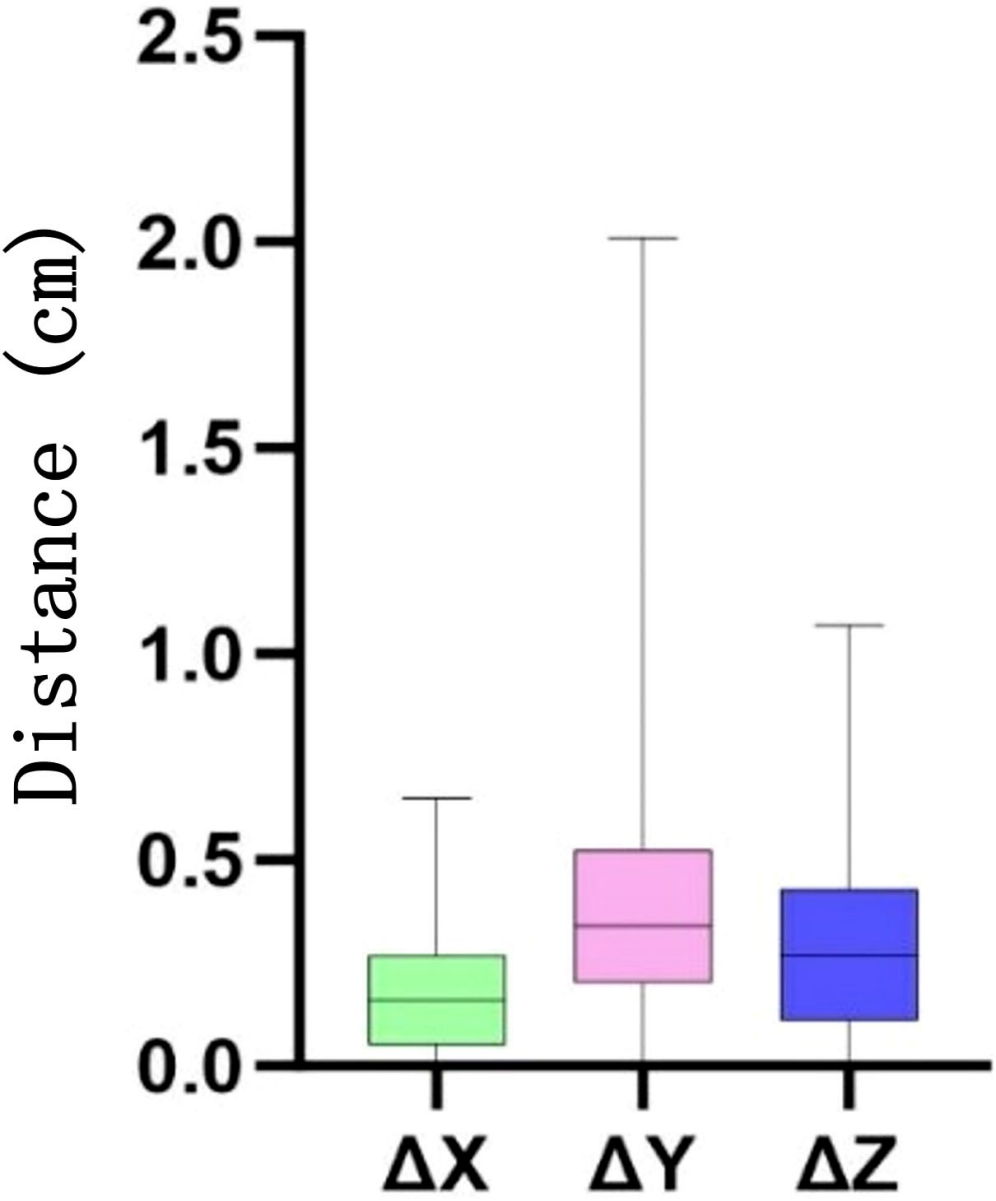
Distance (in centimeters) of the heart movement in the *X*, *Y*, and *Z* directions at different times. Δ denotes the difference in the displacement parameters between cone beam CT (CBCT) and planning CT.

**Figure 4:**
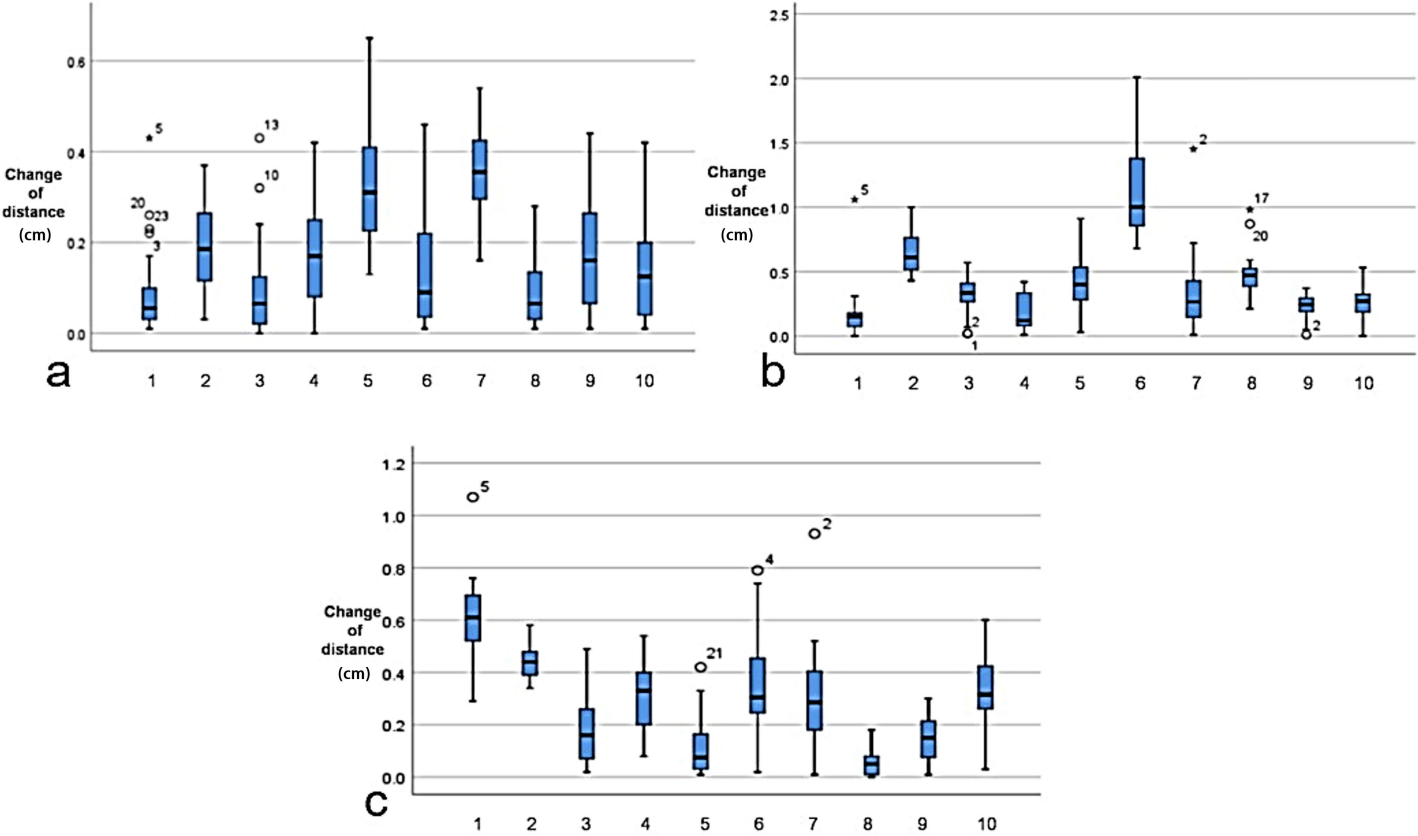
Cardiac displacement variability across orthogonal axes for each patient. **(a)** Left–right (LR/*X*-axis). **(b)** Anterior–posterior (AP/*Y*-axis). **(c)** Superior–inferior (SI/*Z*-axis). *Asterisks* represent extreme values occurring in specific patient–fraction pairs. *Empty circle* denotes potential outliers with positional indexing.

The periodic motion of the heart leads to its displacement. Spatial geometric coordinates of the heart were recorded on the planned CT and various CBCT images from 10 patients, and the displacement distances in the *X*, *Y*, and *Z* directions were calculated (**Figures 3**, **4**) to detect correlations with the dose changes.

For the *X* direction, motion displacement showed weak correlations with the heart *V*_15_ (*r* = 0.14) and *V*_20_ (*r* = 0.14), suggesting that the association between cardiac displacement in this direction and dose is relatively weak within this dose range. A weak negative correlation was observed between motion displacement and *D*_max_ (*r* = −0.20), suggesting that the *X* direction displacement decreases slightly with increasing maximum heart dose. A moderate positive correlation was found with the LAD *D*_max_ (*r* = 0.33), indicating that, to some extent, the dose to the LAD is better associated with the heart’s displacement in the *X* direction (**Figure 2F**).

For the *Y* direction, motion displacement exhibited generally low negative correlations (*r* = −0.42 to −0.13) with several variables, including the heart *V*_15_ (*r* = −0.13), *V*_20_ (*r* = −0.16), and *V*_30_ (*r* = −0.14), suggesting that heart displacement in the *Y* direction shows a slight decrease with increasing heart dose. A relatively stronger negative correlation was observed with LAD *V*_5_ (*r* = −0.42), indicating that this LAD dose exerted a more significant impact on the *Y*-direction displacement relative to the other doses. Other LAD dose variables (i.e., *V*_15_, *V*_20_, and *V*_mean_) also showed negative correlations with motion displacement, implying that, as the LAD dose increases, the heart’s motion displacement in the *Y* direction potentially decreases (**Figure 2G**).

For the *Z* direction, motion displacement showed moderate to strong positive correlations (*r* = 0.38–0.67) with several heart dose indicators: *V*_5_ (*r* = 0.65), *V*_15_ (*r* = 0.62), *V*_20_ (*r* = 0.61), *V*_30_ (*r* = 0.62), *D*_mean_ (*r* = 0.67), and *D*_max_ (*r* = 0.53). These correlations suggest that higher heart doses within these ranges are associated with greater heart displacement in the *Z* direction. Moderate positive correlations were also observed for the LAD *V*_5_ (*r* = 0.38), *V*_15_ (*r* = 0.57), and *V*_20_ (*r* = 0.61), indicating that there is a direct relationship between these LAD doses and the heart motion displacement in the *Z* direction (**Figure 2H**).

Overall, displacement in the anterior–posterior (*Z*) direction demonstrated the most significant impact on dose.

### 3.4 Expanding margins of the heart and the LAD

The average displacement of the heart and the LAD in the *X*, *Y*, and *Z* directions across 25 treatments per patient was calculated as a single systematic error. The standard deviation of this displacement was considered the random error. The overall systematic error (Σ) and the random error (*σ*) for the 10 patients were then determined as the standard deviations of these individual systematic and random errors, respectively. Using McKenzie’s expansion margin formula (*M* = 1.3Σ + 0.5*σ*) (7), the expansion boundaries in the three directions were calculated (**Tables 2**, **3**). In summary, for patients with left-sided breast cancer treated with DIBH radiotherapy, the heart requires planning organ-at-risk volume (PRV) expansions of 1.33 mm (left–right), 4.10 mm (superior–inferior), and 2.42 mm (anterior–posterior). On the other hand, the LAD requires expansions of 3.13 mm (left–right), 1.79 mm (superior–inferior), and 5.43 mm (anterior–posterior) to ensure adequate dose sparing.

**Table 2:** Systematic error (∑, in millimeters), random error (*σ*, in millimeters), and the expansion boundary (*M*, in millimeters) of the heart in three directions (*X*, *Y*, and *Z*).

Error Type	*X*	*Y*	*Z*
∑	0.96	2.87	1.67
***σ***	0.17	0.74	0.51
***M***	1.33	4.10	2.42

**Table 3:** Systematic error (∑, in millimeters), random error (*σ*, in millimeters), and expansion boundary (*M*, in millimeters) for the left anterior descending coronary artery (LAD) in three directions (*X*, *Y*, and *Z*).

Error Type	*X*	*Y*	*Z*
∑	2.10	1.20	3.91
***σ***	0.80	0.47	0.70
***M***	3.13	1.79	5.43

### 3.5 Correlation between distance and dose of the bounding boxes of different sub-hearts

**Figure 5:**
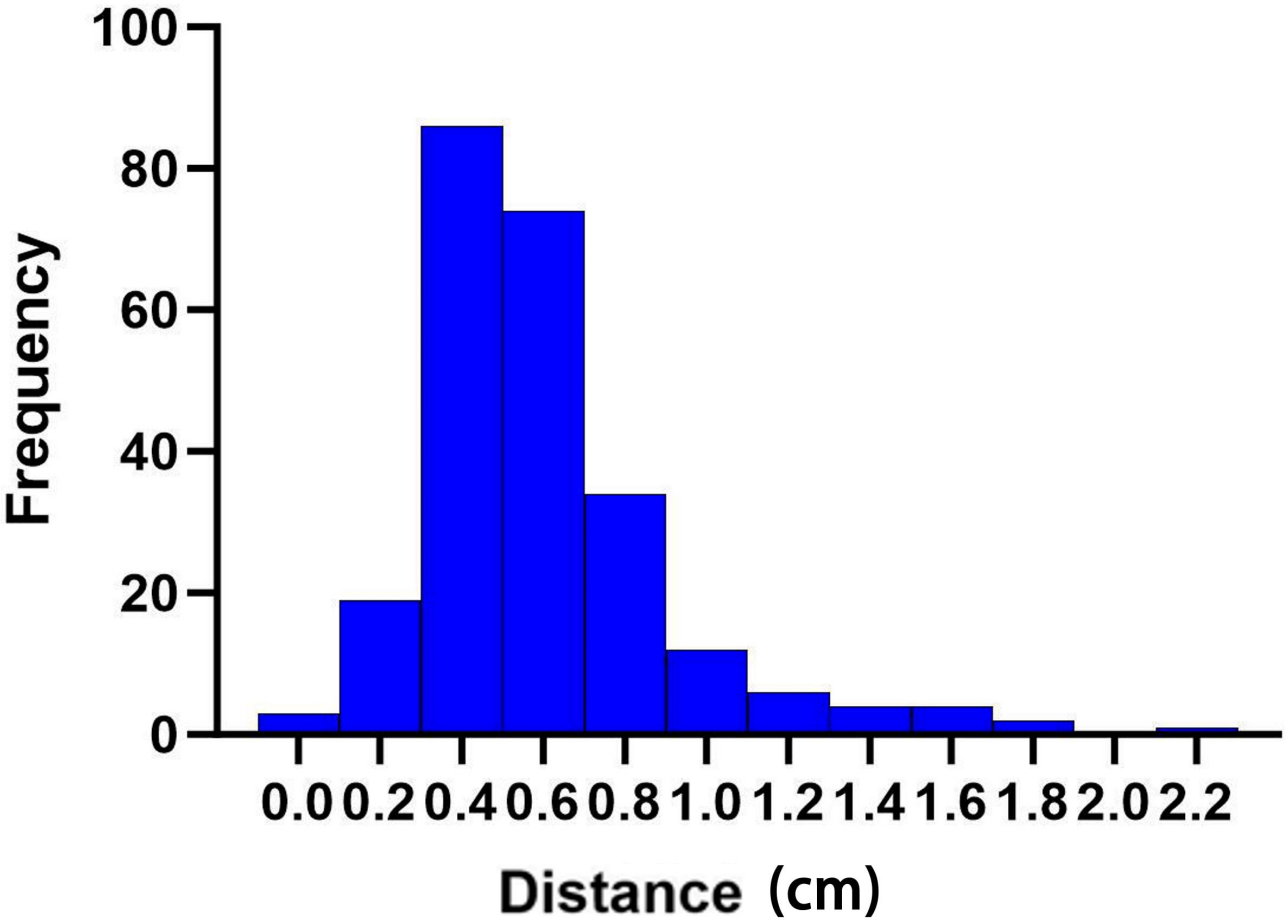
Distance changes (in centimeters) in the cardiac bounding box at different times of cone beam CT (CBCT).

The centroid movement of the heart, represented by the distance of the heart bounding box on different CBCT images across the 10 patients and at different time points, is illustrated in **Figure 5**. Weak positive correlations were observed with the heart *V*_5_ (*r* = 0.30), *V*_15_ (*r* = 0.18), *V*_20_ (*r* = 0.16), *V*_30_ (*r* = 0.18), and *D*_mean_ (*r* = 0.20) (**Figure 2I**). This suggests that as the extent of the heart bounding box increases, certain heart radiation dose/volume indicators also tend to slightly increase. For example, the positive correlation with heart *V*_5_ indicates that a larger heart bounding box might correspond to a greater volume of heart tissue receiving 5 Gy of radiation or more. Conversely, very weak negative correlations were found with the LAD *V*_5_ (*r* = −0.22) and *D*_max_ (*r* = −0.16), suggesting that the heart centroid movement had only a limited impact on these specific LAD dose/volume parameters. Specifically, changes in the size of the heart bounding box do not significantly influence the radiation dose or volume received by the LAD, or larger heart movements do not necessarily lead to higher LAD doses.

## 4 Discussion

As the current standard for setup error correction in radiotherapy workflows, CBCT image registration and alignment with planning CT effectively mitigate positioning discrepancies. Our study was conducted after this correction, and all parameters—including the gating window settings for DIBH—remained identical to those established during the initial simulation. Thus, the cardiac and LAD positional and dosimetric variations observed when DIBH was employed were exclusively attributable to intrinsic cardiac motion. Our department utilizes DIBH to minimize ionizing radiation damage to the heart and the LAD in postoperative left-sided breast cancer patients to avoid dosimetric errors caused by respiratory motion. Regarding the correlation between the cardiac volume and the dosimetric parameters, our findings indicate that, although the cardiac *V*_5_ decreased overall, the apex may be displaced toward high-dose regions in proximity to the target volume (8), consequently increasing *V*_30_. This demonstrates that the cardiac volume alters the spatial distribution of the radiation dose rather than the total integral dose. Concerning the correlation between the cardiac centroid displacement and the dosimetric parameters, dose variations within the dose gradient zones due to cardiac motion align with geometric principles. Superior–inferior (*Y*-axis) displacement moves the heart closer to the chest wall irradiation fields, indicating that DIBH requires stringent control of respiratory fluctuations along this direction. For the anterior–posterior (*Z*-axis) motion, the observed correlation with the LAD dose likely stems from the LAD’s anatomical adherence to the anterior interventricular groove, where positional changes directly modify its distance from the field boundaries.

The association between the cardiac bounding box dimensions and the dosimetric parameters reveals that greater bounding box displacement corresponds to increased dose uncertainty. The negative correlation between the inferior bounding box displacement distance and the LAD dose may have occurred due to the caudal movement of the entire heart displacing the LAD away from the beam isocenter.

These findings provide clinical value by quantifying the relationship between the cardiac displacement magnitude and the dosimetric discrepancies and mitigating the long-term risks of radiation-induced coronary injury.

Our findings emphasized the importance of considering cardiac volume variations when designing and implementing radiation therapy plans to optimize the dose distribution and to protect the heart. While radiation therapy can reduce the 15-year breast cancer mortality by 5% in patients with breast cancer, it also increases the risk of cardiac injury-related mortality (9). The likelihood of coronary artery damage is dependent on the radiation dose. Every 1 Gy increase in the average exposure dose is correlated with a 7.4% rise in the probability of coronary artery disease (10). To enhance the radiotherapy plan accuracy and assess dose instability, in this study, CBCT was used to evaluate the anatomical structure errors and analyze the inter-fraction dose discrepancies. The DIBH technique was employed to mitigate the respiratory motion in left-sided breast cancer radiotherapy. Rigid registration comparisons between the planning CT and multiple pretreatment CBCT images allowed for tracking the positional and dose variations in the heart and the LAD across fractions. Our observation of the inter-fraction displacement affecting the delivered dose aligns with research indicating that a heartbeat exerts a significant impact on cardiac and LAD dose evaluation. Furthermore, it was found that the heart centroid motion influences the dose to both the heart and the LAD, with anterior–posterior displacement exerting the greatest impact. Even after accounting for anatomical positional shifts, cardiac motion still affected the inter-fraction doses to these critical structures. Treatment delivery on the accelerator typically relies on rigid registration between the CBCT and planning CT images. Accordingly, our OAR dose evaluation was based on this method. Although conventional fractionation (e.g., 50 Gy in 25 fractions) is common, a cumulative cardiac dose over the long term can elevate the risk of radiation-induced cardiac injury, particularly in left-sided cases. Hypofractionated regimens (e.g., 42.5 Gy in 16 fractions) offer a potential advantage by reducing the cumulative cardiac dose at equivalent biological doses through shorter treatment times and adjusted fraction sizes. For instance, in the FAST-Forward trial, a 26-Gy/five-fraction regimen demonstrated comparable cardiac toxicity rates to conventional fractionation, but with a significantly lower mean heart dose (*D*_mean_). In patients with left-sided breast cancer receiving hypofractionated radiotherapy, subclinical cardiac events (e.g., elevated NT-proBNP) have been shown to correlate positively with the mean heart dose (11).

Recent advances in precision radiotherapy for breast cancer have introduced multiple technological innovations to minimize cardiac radiation exposure. These cutting-edge approaches include beam angle optimization, the implementation of IMRT, respiratory gating systems, proton beam therapy, and positional modification techniques. Among these, proton therapy has demonstrated particular dosimetric advantages by delivering highly conformal radiation doses to target volumes while maintaining near-zero exit doses to distal tissues through its characteristic Bragg peak effect, thereby substantially reducing cardiac exposure. Therapeutic efficacy can be further enhanced through strategic combination with DIBH techniques. This synergistic approach capitalizes on two distinct mechanisms: the anatomical displacement of the heart posteriorly away from the radiation field during sustained deep inspiration and the precision of proton beam targeting enabled by advanced image guidance systems. Clinical studies suggest that this multimodal strategy may achieve superior cardioprotection compared with conventional photon-based radiotherapy, particularly for patients with left-sided breast cancer and those with preexisting cardiac risk factors (12).

The evolution of radiotherapy technology has led to reduced cardiac doses and improved breast cancer survival rates. However, radiation-induced heart damage remains a concern. Several studies have focused on optimizing cardiac protection and dose assessment. The analysis of Duane et al. on cardiac segment doses from the 2D planning era highlighted dose correlations and variations, supporting dose ranking for clinical evaluation despite limitations in the quantitative dose–response relationships for 2D plans (13). An EORTC trial planning study demonstrated that modern techniques, including hybrid IMRT and proton therapy, significantly reduced cardiac exposure compared with 2D methods, notably decreasing the LAD *V*_5_ from 100% to 20% with IMPT, while improving target coverage (14). Omidi et al. quantified the cardiopulmonary motion effects on the left ventricular doses through 4D-CT/MRI integration, identifying peak doses during expiration and diastole, with significant regional discrepancies in the AHA (American Heart Association) segmental doses. Collectively, these works advanced personalized radiotherapy through refined cardiac substructure dose optimization and motion-compensated precision delivery (15).

The application of DIBH technology effectively mitigated the respiratory motion in left-sided breast cancer radiotherapy. Rigid registration and comparison of the planning CT and pretreatment CBCT images allowed recording and observing the positional and dosimetric changes in the heart and the LAD across multiple fractions. Our findings demonstrated that inter-fraction displacement significantly impacts the received dose. This aligns with the studies by Jensen et al. (16), which showed that DIBH combined with dynamic volumetric rotational intensity-modulated radiotherapy (t-VMAT) reduces the intra-fraction displacement and dosimetric errors, and Lauche et al. (17), which highlighted the significant influence of heartbeat on cardiac and LAD dose assessment. Our observation that the heart centroid motion, particularly the posterior–anterior displacement, affects doses to both structures is consistent with these findings. Furthermore, research (18) supports that the heartbeat significantly impacts the accuracy of dose assessments. These observations suggest that variations in the heart volume during treatment courses can influence the efficacy of radiotherapy. Understanding this relationship is crucial for optimizing treatment plans and minimizing radiation-induced damage. Therefore, clinical practice should consider the impact of heart volume changes on dose distribution to determine the most effective treatment strategies.

Our data indicated that the LAD received a higher dose than the heart, likely due to its anatomical location, which makes it more vulnerable to radiation exposure during left-sided breast cancer radiotherapy. This aligns with the study by Taylor et al. (19), which reported that the LAD received higher doses than the heart and was also more prone to lesions. Thus, the LAD represents a high-risk structure that warrants careful consideration in radiotherapy planning. The research by Liang et al. (18) applied MR cine to reconstruct images under different cardiac cycles. The authors found that, for patients with breast cancer undergoing radiation therapy, increased front–back direction movements can cause an increase in the volume entering or approaching the irradiation range, resulting in a potentially significant dose escalation. Combined with DIBH, cardiac motion analysis revealed significant positional, volumetric, and dosimetric variations in the LAD.

The preceding analysis clearly demonstrates a significant variation in the correlation between the heart’s motion displacement in different directions and the dose indicators, with displacement in the *Z* direction exhibiting the most pronounced positive correlation. This highlights the considerable impact of heart motion in the *Z* direction on the dose distribution and dose–volume relationships during radiation therapy, crucial information for dose planning and cardiac protection strategies. Considering the periodic movement of the heart and the influence of setup errors on dose, Levis et al. (20) explored the safe expansion boundary of the LAD for 5 mm. Our study further emphasized the need for appropriate OAR expansion boundaries, concluding that the heart requires expansions of 1.33, 4.10, and 2.42 mm in the *X*, *Y*, and *Z* directions, respectively, while the LAD requires expansions of 3.13, 1.79, and 5.43 mm in the corresponding directions. The complexity of the heart’s motion, beyond simple 3D movements, and the irregular tubular shape of the LAD, which makes its overall structural quantification challenging, likely contribute to the differing expansion distances observed between the heart and the LAD. Furthermore, the center of mass movement may not fully represent the intricate motion pattern of the LAD. While the study of Bahig et al. (21) on cardiac twisting, that of Taylor et al. (22) suggesting 5–10 mm coronary artery expansion, and that of Levis et al. (20) that used ECG-gated imaging to quantify LAD displacement (2.6 mm *X*, 5.0 mm *Y*, and 6.8 mm *Z*, recommending a 5-mm expansion) provided valuable insights, their results differed from ours, possibly due to the inherent complexity of the cardiac and LAD movements. Future research can delve deeper into these intricate motion patterns.

These findings enhance our understanding of the movement of the heart and the LAD during imaging and exposure to radiation. Further research is warranted to elucidate the underlying reasons for the observed correlations, particularly the only weak and sometimes negative associations with the heart centroid movement. This knowledge is crucial for the optimization of radiation therapy plans and the protection of cardiac tissues. The limitations of our study include its small sample size, which comprised 10 carefully selected patients. While the cohort size appears limited, the substantial longitudinal data collected, which consisted of 25 CBCT scans per patient, yielded 250 volumetric datasets, a statistically robust sample for intra-fraction motion analysis. Based on the anatomical characteristics of mastectomy patients, whose thinner chest walls may amplify cardiac exposure to respiratory motion effects compared with breast-conserving surgery (BCS) recipients, distinct margin adaptation strategies may be required when applying this technique to BCS patients. Other limitations include the blurriness of the CBCT images, which potentially affected registration and delineation, reliance on planned system doses without long-term cardiac damage assessment, and a focus solely on the intra-fraction heart motion, neglecting inter-fraction target contraction. The complex heart and LAD motion may also be inadequately represented by six degrees of freedom. Future studies should employ larger, more representative samples, higher-resolution imaging, long-term follow-up for cardiac damage assessment, and more comprehensive motion analysis methods. Given that the breast size, the distance from the cardiac substructures to the chest wall, the ipsilateral lung volume ratio between the free-breathing and DIBH phases, the body mass index (BMI), and the maximum heart depth (23) are established predictors of cardiac sparing, future studies will stratify primary beneficiaries based on these factors. This prospective stratification will clinically validate dosimetric improvements in targeted cohorts.

In conclusion, a significant relationship exists between the changes in the heart and LAD dose–volume parameters and the cardiac morphological changes in patients with breast cancer undergoing DIBH radiotherapy. Evaluation of the heart and LAD doses based solely on static CT for post-mastectomy left-sided breast cancer patients receiving DIBH can lead to inaccuracies. Therefore, the radiotherapy plan design must consider the periodic motion of the heart and implement personalized target area expansions to ensure safe irradiated doses for these critical structures.

## Data Availability

The raw data supporting the conclusions of this article will be made available by the authors, without undue reservation.
